# Comparative analysis of the *Pocillopora damicornis* genome highlights role of immune system in coral evolution

**DOI:** 10.1038/s41598-018-34459-8

**Published:** 2018-10-31

**Authors:** R. Cunning, R. A. Bay, P. Gillette, A. C. Baker, N. Traylor-Knowles

**Affiliations:** 1 Department of Marine Biology and Ecology, University of Miami Rosenstiel School of Marine and Atmospheric Science, 4600 Rickenbacker Causeway, Miami, FL 33149 USA; 2 Daniel P. Haerther Center for Conservation and Research, John G. Shedd Aquarium, 1200 South Lake Shore Drive, Chicago, IL 60605 USA; 3 Department of Evolution and Ecology, University of California Davis, One Shields Ave, Davis, CA 95616 USA

## Abstract

Comparative analysis of the expanding genomic resources for scleractinian corals may provide insights into the evolution of these organisms, with implications for their continued persistence under global climate change. Here, we sequenced and annotated the genome of *Pocillopora damicornis*, one of the most abundant and widespread corals in the world. We compared this genome, based on protein-coding gene orthology, with other publicly available coral genomes (Cnidaria, Anthozoa, Scleractinia), as well as genomes from other anthozoan groups (Actiniaria, Corallimorpharia), and two basal metazoan outgroup phlya (Porifera, Ctenophora). We found that 46.6% of *P. damicornis* genes had orthologs in all other scleractinians, defining a coral ‘core’ genome enriched in basic housekeeping functions. Of these core genes, 3.7% were unique to scleractinians and were enriched in immune functionality, suggesting an important role of the immune system in coral evolution. Genes occurring only in *P. damicornis* were enriched in cellular signaling and stress response pathways, and we found similar immune-related gene family expansions in each coral species, indicating that immune system diversification may be a prominent feature of scleractinian coral evolution at multiple taxonomic levels. Diversification of the immune gene repertoire may underlie scleractinian adaptations to symbiosis, pathogen interactions, and environmental stress.

## Introduction

Scleractinian corals serve the critical ecological role of building reefs that provide billions of dollars annually in goods and services^1^ and sustain high levels of biodiversity^2^. However, corals are declining rapidly as ocean acidification impairs coral calcification and interferes with metabolism^3^, ocean warming disrupts their symbiosis with photosynthetic dinoflagellates (family Symbiodiniaceae)^4^, and outbreaks of coral disease lead to mortality^5^. As basal metazoans, corals provide a model for studying the evolution of biomineralization^6^, symbiosis^7^, and immunity^8,9^ - key traits which mediate ecological responses to these stressors. Understanding the genomic architecture of these traits is therefore critical to understanding corals’ success over evolutionary time^10^ and under future environmental scenarios. In particular, there is great interest in whether corals possess the genes and genetic variation required to acclimatize and/or adapt to rapid climate change^11–13^. Addressing these questions relies on the growing genomic resources available for corals, and establishes a fundamental role for comparative genomic analysis in these organisms.

Genomic resources for corals have expanded rapidly in recent years, with genomic or transcriptomic information now available for at least 20 coral species^10^. Comparative genomics in corals has identified genes important in biomineralization, symbiosis, and environmental stress response^10^, and highlighted the evolution of specific immune gene repertoires in corals^14,15^ However, complete genome sequences have only been analyzed and compared for two coral species, *Acropora digitifera*^16^ and *Stylophora pistillata*^17^, revealing extensive differences in genomic architecture and content. Therefore, additional complete coral genomes and more comprehensive comparative analysis may be transformative in our understanding of the genomic content and evolutionary history of reef-building corals, as well as the importance of specific gene repertoires and diversification within coral lineages.

Here, we present the genome of *Pocillopora damicornis*, one of the most abundant and widespread reef-building corals in the world^18^. This ecologically important coral is a model species and is commonly used in experimental biology and physiology. It is also the subject of a large body of research on speciation^19–21^, population genetics^22–25^, symbiosis ecology^26–28^, and reproduction^29–31^. Consequently, the *P. damicornis* genome sequence advances a number of fields in biology, ecology, and evolution, and provides a direct foundation for future studies in transcriptomics, population genomics, and functional genomics of corals.

Using the *P. damicornis* genome and other publicly available genomes of cnidarians and basal metazoans, we performed a comparative genomic analysis within the Scleractinia. Using this analysis, we address the following critical questions: (1) which genes are specific to or diversified within the scleractinian lineage, (2) which genes are specific to or diversified within individual scleractinian coral species, and (3) which features distinguish the *P. damicornis* genome from those of other corals. We address these questions based on orthology of protein-coding genes, which generalizes the approaches taken by Bhattacharya *et al*.^10^ and Voolstra *et al*.^17^ to a larger set of complete genomes to describe both shared and unique adaptations in the Scleractinia. In comparing these genomes, we reveal prominent diversification and expansion of immune-related genes, demonstrating that immune pathways are the subject of diverse evolutionary adaptations in corals.

## Results and Discussion

### P. damicornis genome assembly and annotation

The estimated genome size of *P. damicornis* is 349 Mb, smaller than other scleractinian genomes analyzed to date (Table 1). The size of the final assembly produced here was 234 Mb, and likely lacks high-identity repetitive content (estimated ~25% of the genome based on 31-mers) that could not be assembled. Total non-repetitive 31mer content was estimated at 262 Mb and the sum of contigs was 226 Mb, indicating that up to 14% of non-repetitive content may also be missing from the assembly, likely due to high heterozygosity (Dovetail Genomics, personal communication). However, the assembly comprises 96.3% contiguous sequence, and has the highest contig N50 (28.5 kb) of any cnidarian genome assembly (Table 1). We identified 26,077 gene models, which is consistent with the gene content of other scleractinian and cnidarian genomes (Table 1). Among these genes, 59.7% had identifiable homologs (E-value $\le$ 10^−5^) in the SwissProt database, 73% contained identifiable homologs in at least one of the other 10 genomes, and 83.7% contained protein domains annotated by InterProScan. Genome completeness was evaluated using BUSCO, which found that 88.4% of metazoan single-copy orthologs were present and complete (0.5% were duplicated), 2.9% were present but fragmented, and 8.7% were missing. Together, these statistics indicate the *P. damicornis* genome assembly is of high quality and mostly complete (Table 1).

**Table 1:** Assembly and annotation statistics for the *P. damicornis* genome (Pdam) and others used for comparative analysis. (Spis = *Stylophora pistillata*^17^; Adig = *Acropora digitifera*^16^; Ofav = *Orbicella faveolata*^100^; Disc = *Discosoma spp*.^101^; Afen = *Amplexidiscus fenestrafer*^101^; Aipt = *Aiptasia*^85^; Nema = *Nematostella vectensis*^102^; Hydr = *Hydra vulgaris*^103^; Mlei = *Mnemiopsis leidyi*^104^; Aque = *Amphimedon queenslandica*^105^). *Re-annotated using present pipeline (github.com/jrcunning/ofav-genome).

	Pdam	Spis	Adig	Ofav*	Disc	Afen	Aipt	Nema	Hydr	Mlei	Aque
Genome size (Mb)	349†	434	420		428	350	260	329	1300		190
Assembly size (Mb)	234	400	419	486	445	370	258	356	852	156	167
Total contig size (Mb)	226	358	365	356	364	306	213	297	785	150	145
Contig/Assembly (%)	96.3	89.5	87	73.3	81.9	82.6	82.5	83.4	92.2	96.5	86.8
Contig N50 (kb)	25.9	14.9	10.9	7.4	18.7	20.1	14.9	19.8	9.7	11.9	11.2
Scaffold N50 (kb)	326	457	191	1162	770	510	440	472	92.5	187	120
No. gene models	26077	25769^‡^	23668	37660	23199	21372	29269	27273	31452	16554	29867
No. complete gene models	21389	25563^‡^	16434	29679	16082‡	15552‡	26658	13343			
BUSCO completeness (%)	88.4	72.2	34.3	71							
Mean exon length (bp)	245	262^‡^	230	240	226	218	354	208		314	
Mean intron length (bp)	667	917^‡^	952	1146	1119	1047	638	800		898	80
Protein length (mean aa)	455	615^‡^	424	413	450‡	475^‡^	517	331		154	280

^†^Computed as total occurrences for non-error 31-mers divided by homozygous-peak depth (Dovetail Genomics).

^‡^Calculated in this study using GAG based on publicly available data.

### Genomic feature frequency phylogeny

**Figure 1:**
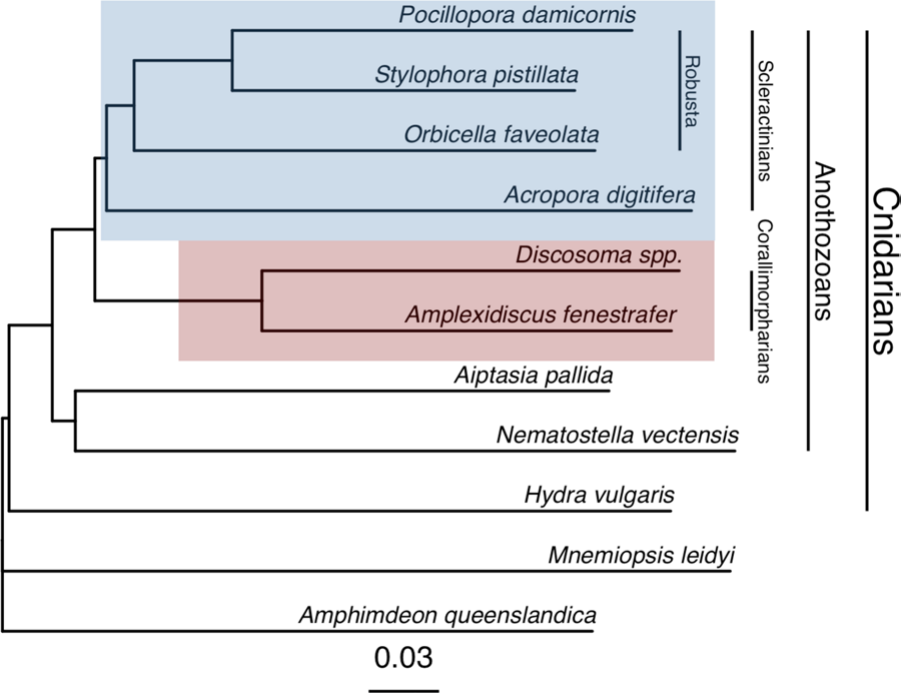
Genome phylogeny. Feature frequency profiling of protein-coding gene models produces a genome scale phylogeny that supports a monophyletic scleractinian clade (blue) with corallimorpharians as a sister clade (red). Bootstrap support from 100 pseudoreplicates was 100% at every node.

Feature frequency profiling shows the phylogenetic relationships among the genomes analyzed here (Fig. 1). This genome-scale analysis resolves the Complexa (*A. digitifera*) and Robusta branches (*P. damicornis, S. pistillata, O. faveolata*) of the scleractinians as a monophyletic sister clade to the corallimorpharians, lending further support to the conclusions of Lin *et al*.^32^ that corallimorpharians are not ‘naked corals’.

### Scleractinian gene content and core function

**Figure 2:**
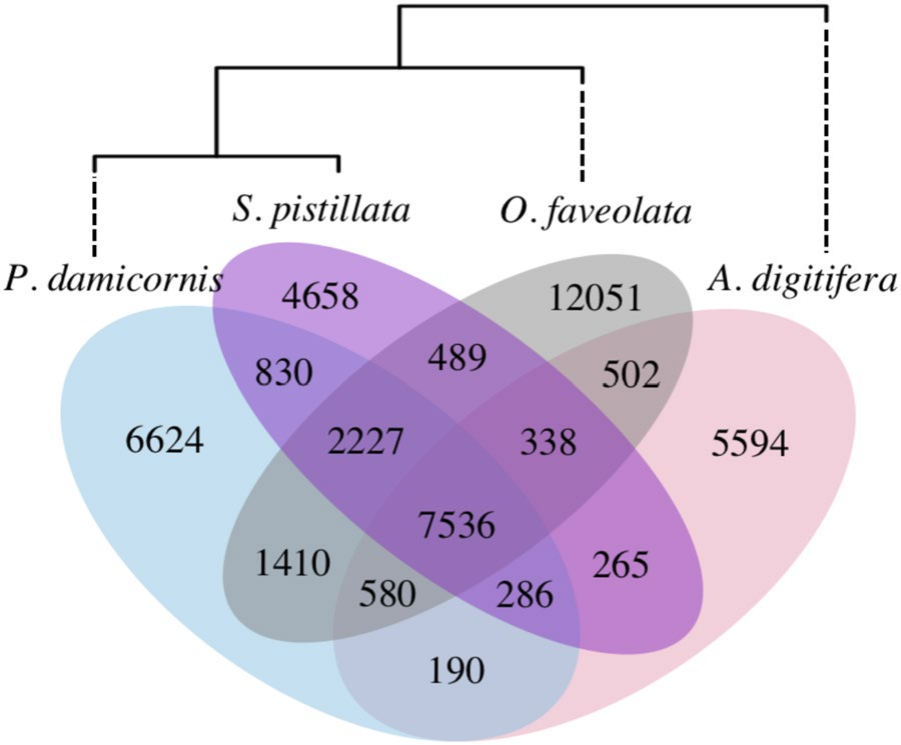
Species-specific and shared gene families across four scleractinian genomes. Numbers indicate gene families, including both single-copy genes and multi-copy gene families. Dendrogram is based on shared gene content, following^33^.

Gene families were identified by ortholog clustering across the 11 genomes in Table 1 (Supplementary Data S1). Across all four scleractinian genomes, we identified 43,580 ortholog groups ranging in size from 1 to 566 genes, with 14,653 of these gene families present in more than one coral species. That only a third of ortholog groups occurred in multiple genomes suggests high divergence among scleractinians, consistent with the findings of Voolstra *et al*.^17^. The highest number of shared ortholog groups occurred between *P. damicornis* and *S. pistillata*, the two most closely related species, and a dendrogram based on shared gene content^33^ reproduces the evolutionary relationships among the four corals^34^ (Fig. 2). Although gene content in *O. faveolata* is more similar overall to the other robust corals than the complex *A. digitifera, O. faveolata* also has the highest number of species-specific gene families, which may reflect its large genome size and/or adaptations to the Atlantic Ocean.

A total of 7,536 ortholog groups were found in all four scleractinian genomes, constituting putative coral ‘core’ genes. Members of these core gene families comprised 46.6% of all *P. damicornis* genes, and functional profiling of the core genome revealed significant enrichment of 44 GO terms associated with basic cellular and metabolic functions, including nucleic acid synthesis and processing, cellular signaling and transport, and lipid, carbohydrate, and protein metabolism (Supplementary Data S2). This basic functionality explains why >30% of these gene families were also found in all other cnidarians, and 96.3% had orthologs in at least one non-coral. This is consistent with the identification of basic housekeeping functions in the core (shared) protein sets in other comparative studies^10,17^.

### Genes specific to and diversified in scleractinians are enriched for immune functionality

**Figure 3:**
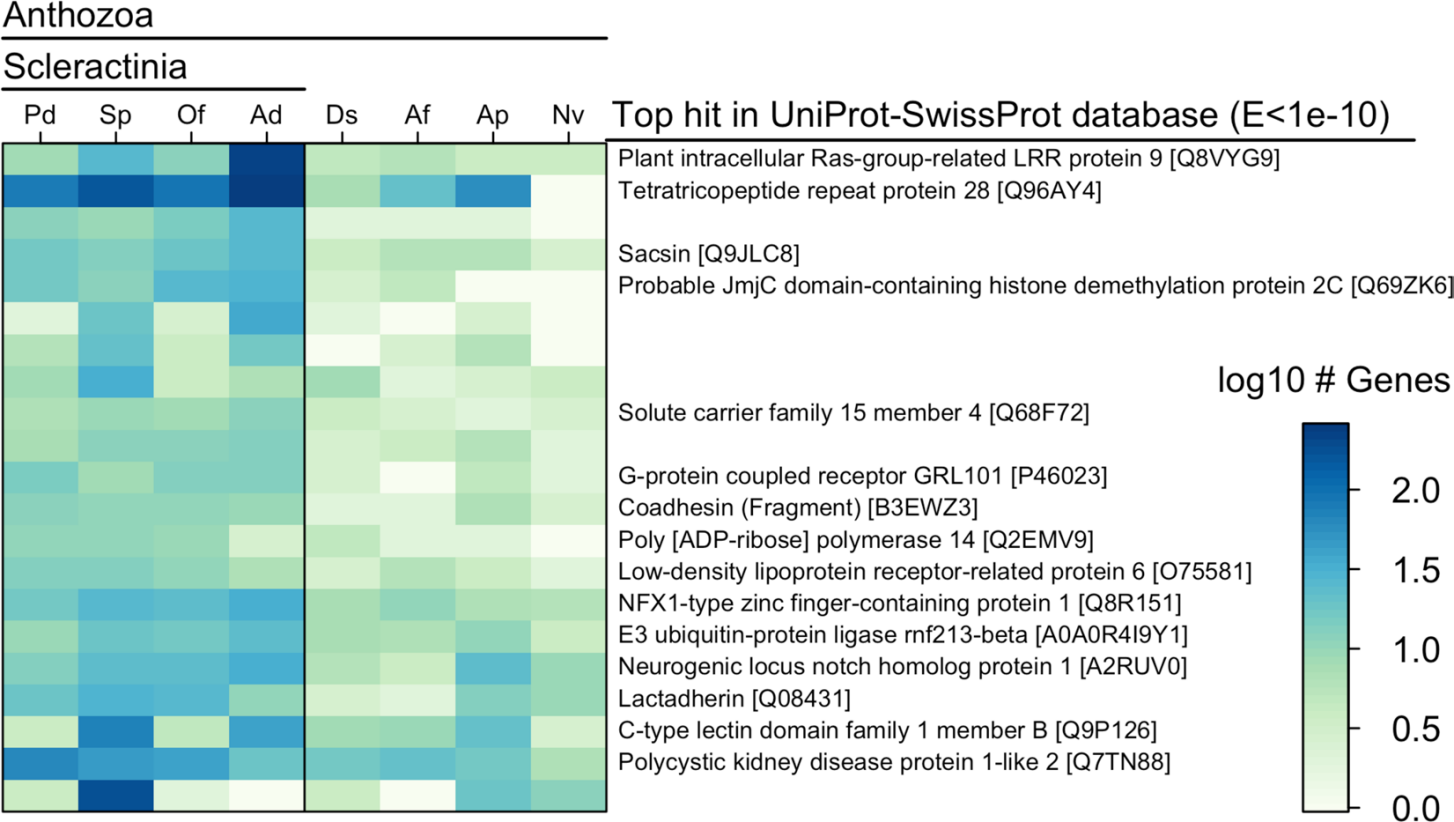
Heatmap showing gene ortholog groups that were larger in scleractinians compared to other cnidarians (Pd = *P. damicornis*, Sp = *S. pistillata*, Of = *O. faveolata*, Ad = *A. digitifera*, Nv = *N. vectensis*, Ap = *A. pallida*, Ds = *Discosoma sp*., Af = *A. fenestrafer*). For each ortholog group, the longest protein sequence from *P. damicornis* was compared to the UniProt-SwissProt database using blastp, and the top hit was selected based on the lowest E-value (if < 1e-10). Uniprot accession numbers are shown in brackets. Sequences with no annotation had no hits to the SwissProt database with E < 1e-10. Gene family sizes and E-values for SwissProt hits can be found in Supplementary Table S1.

A subset of the coral core gene families (n = 278; 3.7%) had no orthologs outside the Scleractinia, suggesting they may reflect important evolutionary innovations within this group^35^. (We refer to these genes as ‘coral-specific’ since they did not have orthologs in the non-scleractinian genomes analyzed here, yet these may still show sequence or domain similarity with genes in other organisms and in reference databases.) Other gene families (n = 21) were significantly larger in scleractinians than other anthozoans (Fisher’s exact test, *p* < 0.01; Fig. 3, Supplementary Table S1), indicating gene family expansion that may underlie adaptation to the scleractinian condition^36,37^. Genes belonging to both coral-specific and coral-diversified families in *P. damicornis* were enriched for GO terms involved in cellular signaling and immunity (Table 2), and showed significant similarity to proteins with known immune function (Fig. 3, Supplementary Table S1).

**Table 2:** Enrichment of GO terms in the coral-specific and coral-diversified genes in *P. damicornis*. Of the coral-specific genes (n = 349), 184 (53%) had GO annotations. Of the coral-diversified genes (n = 339), 229 (68%) had GO annotations.

Gene set	GO Accession	GO Term Name	Observed	Expected	p
Coral-specific	GO:0035434	copper ion transmembrane transport	2	0.10	0.004000
	GO:0007165	signal transduction	32	23.78	0.004200
	GO:0046654	tetrahydrofolate biosynthetic process	1	0.01	0.012300
	GO:0051092	NF-kappaB activation	1	0.02	0.024500
	GO:0070836	caveola assembly	1	0.04	0.036500
	GO:0051607	defense response to virus	2	0.32	0.040300
	GO:0006355	regulation of gene-specific transcription	12	6.06	0.045600
	GO:0000183	chromatin silencing at rDNA	1	0.05	0.048400
	GO:0045132	meiotic chromosome segregation	1	0.05	0.048400
Coral-diversified	GO:0006857	oligopeptide transport	3	0.05	0.000015
	GO:0007264	small GTPase mediated signal transduction	6	1.05	0.000600
	GO:0001822	kidney development	2	0.05	0.000850
	GO:0006468	protein phosphorylation	11	3.82	0.001960
	GO:0007219	Notch signaling pathway	2	0.11	0.004980
	GO:0033151	V(D)J recombination	1	0.01	0.007680
	GO:0045737	positive regulation of CDK activity	1	0.01	0.007680
	GO:0008152	metabolic process	31	30.87	0.012710
	GO:0016255	attachment of GPI anchor to protein	1	0.04	0.037840

Immune-related GO terms that were significantly enriched in the coral-specific gene set included viral defense, signal transduction, and NF-κB pathway regulation (Table 2). NF-κB signaling plays a central role in innate immunity^38,39^, and was recently demonstrated to be conserved and responsive to immune challenge in the coral *O. faveolata*^40^. Signal transduction was associated with 32 coral-specific genes that showed significant similarity to dopamine receptors, neuropeptide receptors, G-protein coupled receptors, and tumor necrosis factor (TNF) receptor-associated factors (TRAFs) (Supplementary Data S3), potentially representing other coral-specific immune pathways. Indeed, the TNF receptor superfamily is more diverse in corals than any organism described thus far other than choanoflagellates^9,41^, with 40 proteins in *A. digitifera*. The *P. damicornis* genome contained 39 proteins with TNFR cysteine-rich domains, suggesting that diversification of this repertoire may be a common feature of corals. Another enriched GO term in the coral-specific gene set was caveola assembly–the formation of structures in cell membranes that anchor transmembrane proteins–which may also play a role in signal transduction and immunity^42^.

The coral-diversified gene families (Fig. 3, Supplementary Table S1) showed high similarity to receptors for pathogen recognition, such as a C-type lectin, G-protein-coupled receptors (GPCRs), and both Notch and Wnt-signaling receptors (lipoprotein receptor-related protein). Notch and Wnt signaling are critical developmental gene pathways with potentially diverse roles in coral biology, but which also have a role in coral innate immunity^43^, particularly in wound-healing processes^39,44^. Other coral-diversified genes were similar to Ras-related proteins with leucine-rich repeats, and a tetratricopeptide repeat-containing protein, which may play roles in signal transduction^45^. Many of these tetratricopeptide repeat proteins also contained a CHAT domain characteristic of caspases^46^, indicating a potential role in apoptotic signaling and/or coral bleaching. Other coral-diversified genes were similar to Poly (ADP-ribose) polymerase, which may act as an anti-apoptotic signal transducer^47^, and lactadherin, which may be involved in phagocytosis and clearance of apoptotic cells^48^. Genes previously found to be differentially expressed in corals under stress or immune challenge were also found in the coral-diversified gene set, including the HSP70 co-chaperone sacsin^49^, the oligopeptide transporter solute carrier family 15^50^, and NFX1-type zinc finger protein^51^. Together, these results suggest that corals as a group have evolved a diverse set of immune signaling genes for interacting with and responding to pathogens and the environment. Importantly, the immune repertoire in corals also contains many other important gene families that are not discussed here (e.g., Toll-like receptors)^39,40^, since we focus only on those that are specific to or diversified in corals.

In addition to defending against pathogens, many of the immune pathways highlighted here may mediate the establishment and maintenance of symbiosis, including with beneficial bacteria and the Symbiodiniaceae^15^. Indeed, lectins and other pattern recognition receptors have previously been shown to regulate symbiont uptake and specificity^7,52,53^, while caspases have previously been shown to mediate bleaching and symbiont removal through apoptosis^54^. We also found that copper ion transmembrane transport was highly enriched in the coral-specific gene set (Table 2), which may reflect an important role for delivery of copper to endosymbionts, where it is a critical component of photosynthetic proteins (plastocyanin) and antioxidants (superoxide dismutase)^55^. For example, in mycorrhizal symbioses, fungi are known to deliver copper to their photosynthetic plant partners^56^, and shortage of trace metals such as copper has recently been linked to coral bleaching^57^.

In addition to immunity and symbiosis, the genes specific to and diversified within corals may underlie other unique scleractinian traits. Calcium carbonate skeleton formation, for example, may be linked to the diversification of calcium ion channels (e.g., polycystins) and cell adhesion proteins (e.g., coadhesin, Fig. 3), which have previously been identified as components of the skeletal organic matrix^6,58^. Corals may also have diversified mechanisms for controlling gene expression, evidenced by the enrichment of transcriptional regulation and chromatin silencing functions in the coral-specific gene set (Table 2), and the diversification of a histone demethylation protein family (Fig. 3). Finally, we note that some enriched GO annotations do not translate directly to corals (e.g., kidney development), and/or are only represented by a single gene (Table 2), and should therefore be interpreted with caution.

### Within-species gene diversification also highlights immune function in scleractinians

**Figure 4:**
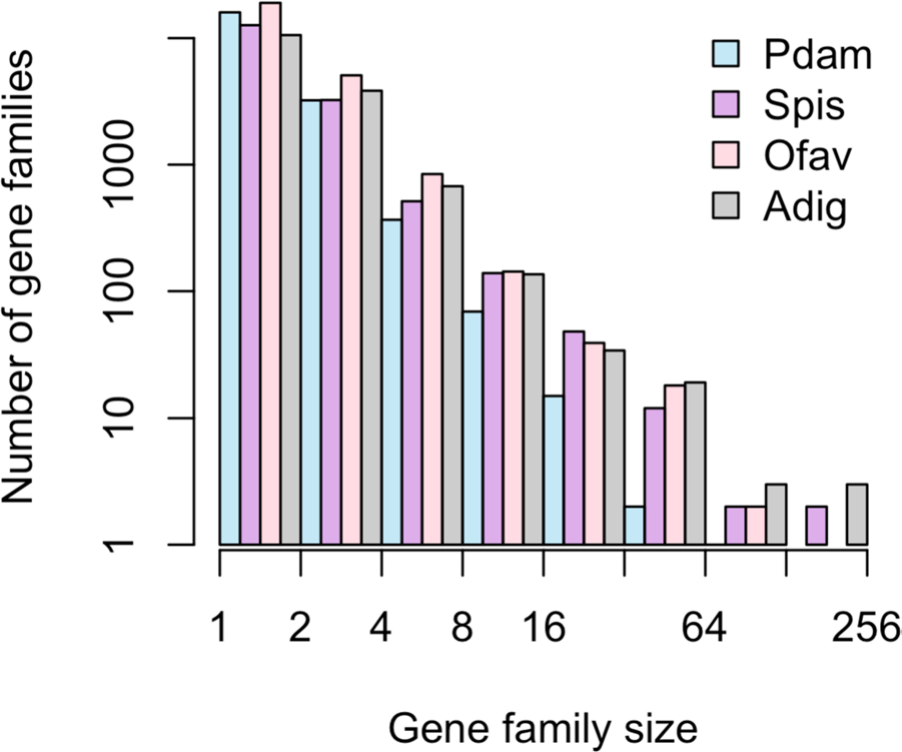
Gene family size distribution in four coral genomes. Pdam = *P. damicornis*, Spis = *S. pistillata*, Ofav = *O. faveolata*, Adig = *A. digitifera*. Bars represent the total number of gene families in a given size class using exponential binning, with each interval open on the left (i.e., the first interval contains gene families of size 1, the second interval contains gene families of size 2 and 3, etc.).

The expansion of gene families within individual lineages may represent an important mechanism of molecular evolution driving adaptation and speciation^36^. Consistent with patterns of gene family size in other organisms^59^, the number of coral gene families decreased exponentially as gene family size increased (Fig. 4). *P. damicornis* had smaller gene families overall, and the fewest large gene families (n = 3 with size > 32, max size = 75), while *A. digitifera* had the most large gene families (n = 25 with size > 32, max size = 255), consistent with pervasive gene duplication in this species suggested by Voolstra *et al*.^17^. However, statistical comparison of shared gene family sizes across the four coral species, accounting for differences in total gene content, indicated that *S. pistillata* had the most significantly expanded gene families (n = 16), followed by *A. digitifera* (n = 11). Even though *O. faveolata* had the highest number of non-shared gene families (Fig. 2), only one shared gene family was significantly expanded, suggesting that its large genome size is the result of species-specific genes and/or even expansion of shared gene content. Finally, *P. damicornis* had no significantly expanded gene families relative to the other scleractinians, confirming that uneven gene family size^17^ and lineage-specific gene family expansion is common in the Scleractinia.

Among the gene families showing lineage-specific expansions in corals, several were similar to reverse transcriptases and transposable elements (Supplementary Table S2); these may represent ‘genetic parasites’ that propagate across the genome^60^, but they may also play crucial roles in genome evolution and the regulation of gene expression^61^. Annotations of other expanded gene families (Supplementary Table S2) suggest important roles in interacting with the environment, cellular signaling, and immunity. While uneven gene family size could also reflect variation in assembly completeness and quality of genome assemblies^62^, these annotations are consistent with categories of genes known to undergo lineage-specific expansion across eukaryotes^45^.

One expanded gene family in *A. digitifera* was similar to NOD-like receptors (NLRs), which are cytoplasmic pattern recognition receptors that play a key role in pathogen detection and immune activation^63^. Characterized by the presence of NACHT domains, NLR genes are highly diversified and variable in number in the genomes of cnidarians^64^ and other species^60^. The expansion of this gene family in *A. digitifera* is consistent with these observations, and may represent adaptation to a new pathogen environment^65^, or to species-specific symbiotic interactions with microbial eukaryotes and prokaryotes^39^. Another expanded gene family in *A. digitifera* was similar to ephrin-like receptors, which may mediate signaling cascades and cell to cell communication^66^. In *S. pistillata*, one expanded gene family was similar to tachylectin-2, a pattern recognition receptor that has been identified in many cnidarians^39^. Previously, a tachylectin-2 homolog was found to be under selection in the coral *Oculina*^67^, providing more evidence that such genes are involved in adaptive evolution in corals. The one significantly expanded gene family in *O. faveolata* did not have a strong hit in the SwissProt database, but did contain a caspase-like domain suggesting a role in apoptosis, which was recently linked to disease susceptibility in this species^68^. Overall, differential expansion of genes related to the immune system is consistent with the findings of Voolstra *et al*.^17^, and suggests that this phenomenon is a general attribute of corals. Lineage-specific immune diversification in corals and other taxa may reflect interactions with specific consortia of eukaryotic and prokaryotic microbial symbionts^69^.

In addition to putative immune-related function, genes that have undergone lineage-specific expansions in corals may also play roles in biomineralization, which could contribute to variation in growth and morphology among coral species. For example, one significantly expanded gene family in *A. digitifera* was similar to a CUB and peptidase domain-containing protein that was found to be secreted in the skeletal organic matrix^58^, and another in *S. pistillata* was similar to fibrillar collagen with roles in biomineralization^70^.

Although the *P. damicornis* genome did not contain any gene families that were significantly expanded relative to the other corals, it did contain many genes (n = 6,966, 26.7%) with no orthologs in other genomes. While most of these *P. damicornis*-specific genes were unannotatable, protein domain homology revealed significant enrichment for 11 GO terms, including G-protein coupled receptor (GPCR) signaling pathway, bioluminescence, activation of NF-κB-inducing kinase, and positive regulation of JNK cascade (Table 3). The mitogen-activated protein kinase JNK plays a role in responses to stress stimuli, inflammation, and apoptosis^71^. JNK prevents the accumulation of reactive oxygen species (ROS) in corals in response to thermal and UV stress, and inhibition of JNK leads to coral bleaching and cell death^72^. The NF-κB transcription factor may also link oxidative stress and apoptosis involved in coral bleaching^73^, in addition to its central role in innate immunity^39^. The occurrence of lineage-specific genes that may function in these pathways indicates that *P. damicornis* may have evolved unique immune strategies for coping with environmental stress.

**Table 3:** Enrichment of GO terms in the *P. damicornis*-specific gene set. This gene set included 6,966 genes, of which 1,498 (22%) had GO annotations.

GO Accession	GO Term Name	Observed	Expected	p
GO:0007186	G-protein coupled receptor signaling pathway	407	149.36	<1e-30
GO:0008218	bioluminescence	15	3.24	6.00E-08
GO:0007250	activation of NF-kappaB-inducing kinase activity	10	3.48	0.0014
GO:0046330	positive regulation of JNK cascade	10	3.48	0.0014
GO:0007165	signal transduction	478	231.92	0.0018
GO:0035278	miRNA mediated inhibition of translation	5	1.68	0.0196
GO:0007411	axon guidance	3	0.72	0.0261
GO:0035385	Roundabout signaling pathway	3	0.72	0.0261
GO:0006814	sodium ion transport	11	6.25	0.0419
GO:0050482	arachidonic acid secretion	5	2.04	0.0446
GO:0015074	DNA integration	11	6.37	0.0474

An expanded role of immunity in *P. damicornis* may explain how *Pocillopora* has achieved such a widespread distribution^18,19^. Indeed, *Pocillopora* corals function as fast-growing and weedy pioneer species in Hawaii^74^, on the Great Barrier Reef^75^, and in the eastern tropical Pacific (ETP)^76^. In fact, in the ETP, where the coral used in this study was collected from, *Pocillopora* thrives in marginal habitats, often dealing with elevated turbidity and reduced salinity after heavy rainfall events, subaerial exposure during extreme low tides, and both warm- and cold-water stress due to ENSO events and periodic upwelling^77^. A diversified immune system may also allow for flexibility in symbiosis, which may further contribute to the success of *Pocillopora*^26,78^. While the wide distribution of *P. damicornis* suggests there may be considerable variation in its genome that is not captured by our sample from the ETP, this work provides a foundation for future genomic analysis in this important coral species.

## Conclusions

This comparative analysis revealed significant expansion of immune-related pathways within the Scleractinia, and further lineage-specific diversification within each scleractinian species. Different immune genes were diversified in each species (e.g., Nod-like and tachylectin-like receptors in *A. digitifera* and *S. pistillata*, and caspase-like and JNK signaling genes in *O. faveolata* and *P. damicornis*), suggesting diverse adaptive roles for innate immune pathways. Indeed, immune pathways govern the interactions between corals and their algal endosymbionts^15,79^, the susceptibility of corals to disease^80^, and their responses to environmental stress^72^. Therefore, prominent diversification of immune-related functionality across the Scleractinia is not surprising, and may underlie responses to selection involving symbiosis, self-defense, and stress-susceptibility.

The function and diversity of both the Scleractinia-specific and the species-specific immune repertoires deserve further study as they could prove to be critical for coral survival in the face of climate change. Indeed, factors placing high selection pressure on corals, such as bleaching and disease, both involve challenges to the immune system. Lineage-specific adaptations indicate corals continue to evolve novel immune-related functionality in response to niche-specific selection pressures. These results suggest that evolution of the innate immune system has been a defining feature of the success of scleractinian corals, and likewise may mediate their continued success under climate change scenarios.

## Methods

### P. damicornis genome sequencing and assembly

The *P. damicornis* genotype used for sequencing was collected from Saboga Is., Panama in March 2005, and cultured indoors at the University of Miami Coral Resource Facility until the time of sampling. Genomic DNA was extracted from two healthy fragments and two bleached fragments of this genotype in September 2016 using a Qiagen DNAeasy Midi kit and shipped overnight on dry ice to Dovetail Genomics (Santa Cruz, CA, USA). The bleached sample (low symbiont load) was used for DNA extraction for shotgun libraries (sequenced on an Illumina HiSeqX) for *de novo* assembly, since this step is more sensitive to contamination. The unbleached sample was used for DNA extraction for Chicago libraries (sequenced on an Illumina HiSeq2500), since this step is more sensitive to DNA size and quality but less sensitive to contamination. Genome scaffolds were assembled *de novo* using the HiRise software pipeline^81^. The Dovetail HiRise scaffolds were then filtered to remove those of potential non-coral origin using BLAST^82^ searches against three databases: (1) Symbiodiniaceae, containing the genomes of *Breviolum minutum*^83^ and *Symbiodinium microadriaticum*^84^, (2) Bacteria, containing 6,954 complete bacterial genomes from NCBI, and (3) viruses, containing 2,996 viral genomes from the phantome database (phantome.org; accessed 2017-03-01). Scaffolds with a BLAST hit to any of these databases with an e-value < 10^−20^ and a bitscore > 1000 were considered to be non-coral in origin and removed from the assembly^85^.

### P. damicornis genome annotation

The filtered assembly was analyzed for completeness using BUSCO^86^ to search for 978 universal metazoan single-copy orthologs. The–long option was passed to BUSCO in order to train the *ab initio* gene prediction software Augustus^87^. Augustus gene prediction parameters were then used in the MAKER pipeline^88^ to annotate gene models, using as supporting evidence two RNA-seq datasets from *P. damicornis*^89,90^, one from closely-related *S. pistillata*^17^, and protein sequences from 20 coral species^10^. Results from this initial MAKER run were used to train a second gene predictor (SNAP)^91^ prior to an iterative MAKER run to refine gene models. Predicted protein sequences were then extracted from the assembly and putative functional annotations were added by searching for homologous proteins in the UniProt Swiss-Prot database^92^ using BLAST (E < 10^−5^), and protein domains using InterProScan^93^. Genome annotation summary statistics were generated using the Genome Annotation Generator software^94^. All data and code to reproduce these annotations as well as subsequent comparative genomic and statistical analyses is available at github.com/jrcunning/pdam-genome.

### Comparative genomic analyses

We compared the predicted protein sequences of four scleractinians, two corallimorpharians, two actiniarians, one hydrozoan, one sponge, and one ctenophore (Table 1), by feature frequency profiling (FFP v3.19)^95^ using features of length 8 to create a whole genome phylogeny for these organisms (Fig. 1). We then identified ortholog groups (gene families) among the predicted proteins from these genomes using the software fastOrtho (http://enews.patricbrc.org/fastortho/) based on the MCL algorithm with a blastp E-value cutoff of 10^−5^. Based on these orthologous gene families, we defined and extracted several gene sets of interest: (1) gene families that were shared by all four scleractinians (i.e., coral ‘core’ genes), (2) gene families that were present in all four scleractinians but absent from other organisms (i.e., coral-specific genes), (3) gene families that were significantly larger in scleractinians relative to other anthozoans (Binomial generalized linear model, FDR-adjusted *p* < 0.01; i.e., coral-diversified genes), (4) gene families that were significantly larger in each scleractinian species relative to other scleractinians (pairwise comparisons using Fisher’s exact test, FDR-adjusted *p* < 0.01; i.e., coral species-specific gene family expansions), and (5) genes present in *P. damicornis* with no orthologs in any other genome (i.e., *P. damicornis*-specific genes).

### Functional characterization

Putative gene functionality was characterized using Gene Ontology (GO) analysis. GO terms were assigned to predicted *P. damicornis* protein sequences using InterProScan^96^. Significantly enriched GO terms in gene sets of interest relative to the whole genome were identified using the R package topGO^97,98^. These analyses were implemented using custom scripts available in the accompanying data repository (github.com/jrcunning/pdam-genome).

## Electronic supplementary material

Supplementary Information

Supplementary Data S1

Supplementary Data S2

Supplementary Data S3

Supplementary Table S1

Supplementary Table S2

## Data Availability

All data and code to reproduce the analyses and figures described herein can be found at github.com/jrcunning/pdam-genome. The full genome assembly and annotation is archived and publicly available at NCBI (BioProject PRJNA454489; BioSample SAMN09007954; Genome RCHS00000000), and in the reefgenomics database^99^ (http://pdam.reefgenomics.org/).
