# Supplementary material for: Comparative analysis of the Pocillopora damicornis genome highlights role of immune system in coral evolution
Source: Sci Rep. 2018 Oct 31;8:16134. doi: 10.1038/s41598-018-34459-8 (PMC6208414; doi:10.1038/s41598-018-34459-8)
Supplement: Supplementary file 1 — Supplementary Information [file 41598_2018_34459_MOESM1_ESM.docx]

Supplementary Information

Comparative analysis of the *Pocillopora damicornis* genome highlights role of immune system in coral evolution

R. Cunning^1,2*^, R. A. Bay^3^, P. Gillette^1^, A.C. Baker^1^, and N. Traylor-Knowles^1*^

^1^Department of Marine Biology and Ecology, University of Miami Rosenstiel School of Marine and Atmospheric Science, 4600 Rickenbacker Causeway, Miami, FL 33149, USA

^2^Daniel P. Haerther Center for Conservation and Research, John G. Shedd Aquarium, 1200 South Lake Shore Drive, Chicago, IL 60605, USA

^3^Department of Evolution and Ecology, University of California Davis, One Shields Ave, Davis, CA 95616, USA

*Correspondence: Ross Cunning (ross.cunning@gmail.com), Nikki Traylor-Knowles (ntraylorknowles@rsmas.miami.edu)

**Supplementary Data and Table legends**

**Supplementary Data S1.** Ortholog clusters across the 11 genomes. Columns indicate 1) the name of the ortholog group, 2) the number of genes in the group, 3) the number of taxa with genes in the group, and 4) names of all genes in the group.

**Supplementary Data S2.** Gene Ontology (GO) term enrichment within the coral ‘core’ genes in *P. damicornis*, relative to the entire genome. Columns indicate 1) the GO term ID, 2) the name of the GO term, 3) the number of times this GO term was observed among the coral core genes, 4) the number of times this term was expected based on its occurrence in the whole genome, and 5) the p-value from the enrichment test in the R package topGO.

**Supplementary Data S3.** Amino acid sequences and SwissProt annotations of the 32 coral-specific genes in *P. damicornis* associated with the GO term ‘signal transduction’, in FASTA format.

**Supplementary Table S1.** Gene families that are larger in corals relative to other anthozoans. Columns indicate (1) the gene family name (i.e., ortholog group), (2-5) the gene family size in the four corals, (6-9) the gene family size in other anthozoans, (10) the p-value from Fisher’s exact test for larger size in corals, and (11) the representative (i.e., longest) gene from *P. damicornis* in this family along with (12) the E-value and (13) top SwissProt annotation for this gene.

**Supplementary Table S2.** Gene families that are larger in one specific coral species relative to the other three. Columns indicate (1) the gene family name (i.e., ortholog group), (2-5) the gene family size in the four coral species, (6) the p-value for differences in size among corals (subsequent pairwise comparisons not shown in table, see R code), and (7) the representative (i.e., longest) gene from the genome in which the gene family is largest along with (12) the top SwissProt annotation for this gene and (13) E-value for this hit.
