# Supplementary material for: Comparative analysis of the Pocillopora damicornis genome highlights role of immune system in coral evolution
Source: Sci Rep. 2018 Oct 31;8:16134. doi: 10.1038/s41598-018-34459-8 (PMC6208414; doi:10.1038/s41598-018-34459-8)
Supplement: Supplementary file 4 — Supplementary Data S3 [file 41598_2018_34459_MOESM4_ESM.docx]

>pdam_00011670-RA protein Name:"Protein of unknown function" AED:0.25 eAED:0.25 QI:78|0.83|0.71|1|0.66|0.42|7|429|551

MESITTTCLIDLAFDLSVDWKTLARVLGLSEEVSRICDDYKRNVFEQAYRMLQTWKRRNGSQATYQALGEALSHDVVSRNDLAQKYCAGRNRSSLDEGIDLKGLKTGMVTIADMVSIANNFGKEWMWVGRLLGLEDSLLDGIKEDLSQTYECTYKMLEWWCKKKDSDATYECLARALLHRTVGMREVAEKFCVDHREKKIASAAPSGEGNTPIDKKLKNLTLNGSTACEFAGSYTEAPGATGNKSLAGTTPPRVPEPADDCITKNVITILPYKVWKELAIKLDPERPLGGDYKDLACEMGYTVEKILYFQSRNGPTEALLTDCANSVSIDELCNKLEAIGRSDAKIVVDEWIKKKTTNSITTTCLIDLAFDLSDDWKKLAHVLGLSGEVSRICDDYRRNVFEQAYRMLQTWKGKKGSQATYQVLREALAHEAVLRNDLVQEYFEGRDRSCLDEEIDLEGLKPGVVSSSDLLSIAKDLGLKWVWIGRLLGLEDSLLDGIREDHIQVSDCQYKMLELWRREKTTGATYQCLARALLHRTVCMRDVAEKFCVEN

>pdam_00022225-RA protein Name:"Similar to D(1)-like dopamine receptor (Oreochromis mossambicus)" AED:0.23 eAED:0.23 QI:0|-1|0|1|-1|1|1|0|357

MNSSRADNNFIPPECSVYKTVGGKLSDSDTQARNVLIFIVITNIVISPLTATFNMLSMIAVKTKRRLREHKSNILLACLAVTDLAVGVIVQPMLTVVSIIDLVGEITRELCLLQICTQFMTSFLVNSSLIHLALVSGERYLATKHVYAYANGLVTEVRLLVASGLAWLLSLILHIPLFVKISVFYATNNIFIGISLAIITSCHVTVYREVRRQQKTLSTQQVTEEARQKFLKDKKVFKLTATIVAVLLFCYLPITVVRIVLNWSGLMSIKVRFTILFFSVSFTLFNSFLNPLIYAIRIRQFRVAFIELLCRTTNLAEAEEMERNMFRRRNAVAVMDRGQKQEEEHHDEGQQISNLST

>pdam_00010427-RA protein Name:"Similar to Adrb1 Beta-1 adrenergic receptor (Rattus norvegicus)" AED:0.28 eAED:0.28 QI:0|-1|0|1|-1|1|1|0|326

MEENQLVYISNVMTCIFNALFAVPAIVGNILVVLAIWKTHLARSPSNILLSTLAFADLQVGLIVQTLFVCHKMAEIFKAEELSCYARFVYIVFGYCITAVSLLTLTAIALERFLALHLHLRYKEVVTKKRILIAACSFWALGIVVTSLYFIQRSVFGSIVIASEVLSIVITSVAYIKIYKIVRRHAREIQCQRRFDLGTQVELNMKKYQRSTFTMVIVFMLSFVCYVPYSLVMVAKLKYGFTVPVKVATDVFSTVVCINSSLNPFIYCWRMKEIKQAVWAVIKRNTGVNPGATEQSTMTFNPLPPKRLDYGSGCKTVPEAPHESVK

>pdam_00003150-RA protein Name:"Similar to SSTR5 Somatostatin receptor type 5 (Homo sapiens)" AED:0.12 eAED:0.14 QI:47|0|0|1|0|0|2|0|613

MILSLIVKLTLQVIVTLFGVFGNLMVVFVFGKFVKKKTSADFYLQNLAIADLGMLLFVFPLLVIRMELPTRWPFGRFSCLYLSSLPEAFYGASVWCITVIAIERYRKIVFLRKPKEKISKTPLKRARIIAFFMWMMSLLVFSLPLNFVVKYRELPTGGKWCGPTWPSLLLLQVYVVILTLLSYVLPLAIIYWTYLAISRKLKQSSSFLRNFNRNQETSSQTKLEALRLRQNKRAKRILTPIVVVFGVTMLPITIIRLIFISWPALTVQKYYENLMFTVTFNTSSEFITSKLSRSSIIKLSVEVFIALFGIFGNVLVAVVVKGLGKKKTATDFYLLNLAIADLGILLISFPLVAIKENAPTNWPLGEFACLYSSPLPEIFHGTSVWFIVVIAIVRYRKIVIPRKAIKNRNKFSLKYTKTIAGCIWVTSFLVFSLPLYFVVSFGLPHGGAAKWCGPIWPSLFFAQLYLCLMTTFGYIIPLGVICWTYLAISRAINHSSNFLYAMKRGQNGAEANLQDAVTSVQSIRLRQNKRAKKILTPVVVVFAITMFPLTILRLTLAFWPSLATQDYYENTLFAVTLFVITNSSANPVIYSIASKSFRKGMTNLYRKCLMKCL

>pdam_00023363-RA protein Name:"Similar to DZIP3 E3 ubiquitin-protein ligase DZIP3 (Homo sapiens)" AED:0.03 eAED:0.03 QI:0|-1|0|1|-1|1|1|0|525

MAAATPSSLASSVEKTNGAKLSRLLIDGGTAVLRKCFNTFHPPTKLAAGLSSHFTTLHTLFKKKVLRLAQWDQLFPPSGDPPDSKEFDITLLFLLLTNMCGLTPPSSGWHAMPPISDTSFEANLARMKFFRNKLYGHVSTTGVEMSVFLSLWRDISAVLVDLGFDQVEIDRLEVEHSGEEDYIDLLREWSESEEDTWSQLRDIRNFQIQMHEDVADLRQNQKEDQKTLEDTKSKLEKLSHCQAKTLEAVEEMQVGIEEFKQVAEYEKKKREDHWEVEALKNLAKVDFRGDIEYHAQRFQEGTREWIFRQMDEWLDDKSSENRVMVISGNAGMGKSVISAVACKRMQKAGRLSGSHFCQHNNVRYRNPQLMLQSLACHLTHTLPEYKEALVEKLSRNLGVPLNSMGVEELFALLLKEPLNAVKDPGRNILMVIDGLDESEYQGRNELLDVVGKHFFKLPKWIRFLVTARPEINITESLKHLQPIHLNQKQEENLSDIKRFFQLRLGKQLEQEHKNVLLDKLVQRTE

>pdam_00023362-RA protein Name:"Similar to DZIP3 E3 ubiquitin-protein ligase DZIP3 (Homo sapiens)" AED:0.47 eAED:0.47 QI:0|0|0|1|0|0|2|0|351

MAAATPSSLASSVEKTNGAKLCRLLIDGGTAVLRKWFNTFHPPSKLAAGLSSHFTTLHTLFKKKVLRLAQWDQLFPPNGDPADSKEFDITLLFLLLTNMYGLTPPSSGWHAMPPVGDTSFEANLARVKFFRNELYGHVSTTGVEMSVFLSLWQEIRAVLVDLGFDQVEIDRLEAEHSGEEDCIDLLREWSESEEDTKSQLRDIRNFQIQMREDVADLRQNQIEDQKILEDTRFKLDKLSQCQAKTLEAVEEMQVGIEEFKQEAEALKNLAKVDFREDIEYHAQRFQEGTREWIFQKIDEWLDDKSSENRVMVISGNAGMGKSVISAVACKRMQEAGRLSGSHFCQHNNVRY

>pdam_00005661-RA protein Name:"Similar to GALR2 Galanin receptor type 2 (Homo sapiens)" AED:0.17 eAED:0.17 QI:0|-1|0|1|-1|1|1|0|300

MRSPINYLLINLATADMIVALFIGIPFVVTPILTHPEGKPGDILCKLFTGGNVGWVGALASVFTLVAVAIERYGAVLYPHSQKGKLTRTRLTVLIATCWTLSALWAIPGFNAITYMPEIKSCAHSWSKPIYAQFYTVGWSVVAGVIPIGILGALYSRMVYHLWLRKDNIREATQKALKRHRKRVTKMVVVVTVIYVLCWVPELLIYFLGFTGTITLVGVHHAIASALIVFNSSVNPIVYSLQSSQFRKHLTDLIFCKRNRVAPMSSTATASTGKRQGLEMEGETNMTVHPSQDVESVTVT

>pdam_00023010-RA protein Name:"Similar to SIFaR Neuropeptide SIFamide receptor (Drosophila melanogaster)" AED:0.32 eAED:0.38 QI:0|0|0|0.66|1|1|3|0|254

MTVNTTVRHFFENSSQSTSHAASSHSDQACEITADSALEVTAKALAYIFIFLVSFFGNIFLLVVIYKKKQLRRSINYFVFNMAVSDLFNPLTIMTVKIVEIISGSSSWKVDRPWLLGNILCKLAYFLPDVSSVVSIGSLLLISIDRLIAVVFPLRAKLISSKVRLISILSTWFVAIAIHAPYFYSFKLIPHENETYCELNWGPAFDHMKTHRGFVTANFITFIPIPICVLATRTTATALGNVFIVIASVAEYQV

>pdam_00023007-RA protein Name:"Similar to Npffr2 Neuropeptide FF receptor 2 (Rattus norvegicus)" AED:0.30 eAED:0.30 QI:0|0|0|1|0|0|3|0|660

MTVKTTVRHFFENSSQSTSQACEIKADSALEVTAKALAYIFIFLVSFFGNIFLLVVIYKKKQLRRSINYFVFNMAVSDLFNPLTIMTVKIVEIISGSSSWKVDRPWLLGNILCKLAYFLPDVSVVVSIGSLLLISIDRLIAVVFPLKAKLISSKVRLISILSTWFVAIAVHAPYFYSFKLILHENKTYCKLNWGPAFDHMKTHRGFVTATFITFILFPICVLAGMYSIIVWTIRKKNKKTKEKFSFRQNHRDQQLRKIVRLAMAIMISFVSCMTPLLIYSFLTNFLWNWESPPICAFQTVLPFISVFLLHSWSAVNPCICFIFSKNYRNGLRQCFHCNCLNRRISGLQDNYQMQTMLPSDPFNSQTTNHTVSSNSDQACEITADSAVEVTAKALAFIFIFLVSFIGNIFILVVIYKNKQLRRSINCFVFNMAVSDLGIPLTRMTVMIVEIFSGSSSWKVDRPWLLGNILCKLAYFLSDVSFVVSIESLLLISMDSLIAVVFPLKAKLISSKTYCLLNWGPAFDHKKTHRGFVTATFIAFIPIPICFLAVVYSITTWKIRKKNEKSKERFSCLQNRRDQQLRKIVRLAMAFMISFVSCMIPLIVIFVWNAIEQPSFCALQRVFPFISVFILYSWSALNPCICFIFSKNYRDGLKQCFHCNG

>pdam_00023006-RA protein Name:"Similar to SIFaR Neuropeptide SIFamide receptor (Drosophila melanogaster)" AED:0.35 eAED:0.47 QI:0|0|0|1|0|0|6|0|496

MKAMTTSYRIRILESRQFVAAGKYSMKTCLLSPRCVYCGFYRKPSFDLDRQALRCRLSTEGQTYQLKSKIDQHSKHVMTSDSAAEITAKAVAYIFIFLVSFFGNIFILMVIYKNKQLRRSINYFVFNMAISDLFNPLTVMTVKIVEIISGSESWKVDRPWLLANILCKLAYFLPDVSLVVSIGSLLLISIDRLIAVVFPLKAKLISSKVRLISILSTWFVAIAVHAPYFYSFKLIPYENETYCIMSWEPAFDHMKTHKGFISASFITFFLIPVVFLVIVYGIIAWTIRKKNKKSKERLSCRQNHRDQQLRKIQELPQRPQAMFTLGRCQSKTTGHTDSSNSDQACEITADSAVEITAKALAYIFIFLVSFFGNIFLLVVIYKNKQLRRSINYFVFNMAVSDLFNPLTVMTVKIVEIISGSSSWKVDRPWLLGNILCKLAYFLPDVSLVVSIGSLLLISIDRLIAVLNWGLAFDHIETHRGFVTATLYRHAYMHVCI

>pdam_00020794-RA protein Name:"Similar to gpr54 G-protein coupled receptor 54 (Oreochromis niloticus)" AED:0.02 eAED:0.02 QI:0|0|0|1|0|0|2|0|652

MNGTKNNSVQDCFGEPSRATTAFKALAYVCIFVVSLIGNGGILLAIYKNKQLRRSINYFVFNMAVSDILNPLTILPVTFVEIVSGSDTWKVDDPWLLGNFLCKLSFFLPDVSVAVSIESLLLISLDRLIAVVFPLRTRYLSPKVHLMSILCAWIIAIAVHAPYFYTFRLQLYENKTYCVANWGPTFNHVETHKRFVTATFITFVLVPICVLVIVYSVIAWSLCQERKKMEQESSNHQKKLGKQSKKIVCSSVAIVAAFAFCMIPQLVFIFTRIFVWNWEVPPNCAFRTTIPFIASFMLHAWSAVNPCICFIFIKTYRDTLKKLLCSLWSTAEKAWKAIAYSLILVVSFVGNVLVLLIFYKNRQLRRSPINFFVFNMAFSDLFNPFTIMPITVVQIISGSDSWKVSNPWLLGNILCKLCYFLPDVSLIVSIQTLLLISFDRLLAVVFPFKASLISPKVRLTAISCTWLIASALHAPYFYTFQLFSDQNEAYCKQLWEPTIADHLETHKKFVTATYIIFFLVPFCLLVFVYGIIAWTLSNNNKRRQRELSCIQRQRDQKLRKTIRMSAAIIIAFFICTTPLFVYMFTMIFIWNWTDDLQACSFHTWIPFFSFFMVHAWSAVNPCICLICINVYRSSLVRILPFSSNVVHVTATR

>pdam_00001905-RA protein Name:"Similar to dl D(5)-like dopamine receptor (Takifugu rubripes)" AED:0.37 eAED:0.37 QI:0|-1|0|1|-1|1|1|0|351

MPFIKNLCEDTRPPYSLSMFTASVSLVLCLITVPGNVLVCLAIFKDPYKELRTSFNFFVFQLAISDLVVGVLTEPMFICFHIREAMKYPVMENIWVIHLTFFISYAASLLSLAALTLDRYLTVMSHHRRLLSTTQITLISVLIWVISFSLPCLYFVTGFYLFAFMFTNASLIIIISILTFSYIRIHQRLKDQISRWHWFKQTQIKLTAMSLEKKLTRAFYIILGIFLMCLLPSFVMIYIIIFCDECDCTLVHWMRDLQFIFLLLNCSLNQFLYAWRARNFRKAISAVFCKTVSYQVNAELAGNNNGIIAADSNSADVIELIPLDPTKLSSMPIEEMGQTNQFSFQSSEKRN

>pdam_00022374-RA protein Name:"Similar to Npffr2 Neuropeptide FF receptor 2 (Rattus norvegicus)" AED:0.27 eAED:0.27 QI:0|0|0|0.5|1|1|2|0|302

MESFDKNISYPRYVAPNITNSTCVAGDSTVEMVAKASAYSTILVFSLVGNAILILSIAKNKQSRKSIHFLVFNMAVSDLFNPFTLMPIHLVQIISGSVSWKVVRPWLLGSILCKLSYFLIDVSLVVSIESLLLISVDRFIAVLFPLKAKLISSKVRLVGVLCTWIVAIMVHAPYFYAYKLTPEGNEVVCRLNWGPAFVHKQTHKRFVTATFITFVLVPIGVMAIVYGTIAWKLKRSNTKQKQQLGCRHKSRDEQHKKIVRMSVAIIVAFSFCTIPLSVYLFAQIVLWDKGLPPVCAFQTRHL

>pdam_00014026-RA protein Name:"Similar to Traf4 TNF receptor-associated factor 4 (Mus musculus)" AED:0.16 eAED:0.16 QI:28|1|1|1|0.85|1|8|0|384

MEDGYSSEEEPESGGHDFVFVDELSPGQTCPICLVAMCNPVQTVCGHRFCESCLQGTFREGAGRVCPQDRISIPEDGGYFRDVAWERDILSLRVKCKMSERGCDWTGMLRYYEDHFQLCEYEDVFCDDCNEELQRRSLNTHQTSECHNRIVQCEHCAMEFAFRLTESHEYECLRWPLDCPQECGILGIPREEVESHVMNDCMMTMVLCPYEEAGCNFYDERSNLKAHIDASREEHLSKTWSKLLKTTERVNELEQVKLHMQTDIDTVKKSLEEAKEDVAQLKLSEAERKLENLKLKKDVLELQMKLKDSHHNVNLPRKQDFDRCIKIEELSCSDTEEDLKDEETTKSSKATKYYLSKPVVGRSKPNVIPKLNPKGAARKVSSRN

>pdam_00006281-RA protein Name:"Similar to ADRB2 Beta-2 adrenergic receptor (Tscherskia triton)" AED:0.04 eAED:0.04 QI:0|0|0|1|0|0.5|2|0|335

MVPLITFLYAFLSILITFANAFVISAVYSTRALRRITNYSLVSLAIADLVVGMVVLPVRICEAQDFRKSQNFSWCQFSLSLTLLSLSASVLNLLIVTVERYFAIILPLSYSSKVTARRNFYAIILVWFVAMFTSFLPFVTLRNMTAKERGREHKICRFADTMSPEYLTFFSAAIVLIPTLFISAAYLKIYRAAVKLRGRLKALQVQRDGNHNIAKALKESKAARTVGIVVGVFYLCWIPFMVAVILSAFVKNLITPVVVLVISVLIYSNSAINPVLYGYLNRDFRLAYKRMFSRISLPSACARVHPRERRRCYELPELSTSSQPGQGIYISDSAL

>pdam_00007753-RA protein Name:"Similar to TkR86C Tachykinin-like peptides receptor 86C (Drosophila melanogaster)" AED:0.10 eAED:0.10 QI:0|0|0|1|0|0.5|2|0|724

MNNTSTTDQFLPQSNPGMTTVKRLLPISIAIIFSNGLVFALFCKRKSLRTSSNYLLLGLAICDFLTGAVNIPYFIVFSFQVVPLNMQKNYNYWLVIIHNFMAVTAAYHLLVITAEKFLAITKPLKHYLVTKKTVLKSLAAIWISSTVIASIPLAWKNSQMQLLFSVIYFSVCLVFVFVIPYAFMIYAFVVMFKAITSKRRPSSIPRRVASGLTWGIVNDRKCILIFALMAAIYVICWLPYFTIGLVIGIKNYLCMELTQPVNKATEIIVLVRYITSFTNPLLYTFFKRDFWRTLRKISHKKEFIHGRSKSSKQREWFLRKKSVENSTTLSRLSWITAEAKDASNVKGSISEEQILFVSSYRLRRLLDMSNSTASDQSSPESNVGMTVLSRLLPIAIAIILCNGLVFVLFCRKKCLRTSSNYLLLGLAICDFLTGAVNIPYFVIFHFPVVPPSMRADFNYWLFIVHTLTAVSAAYHLFIITAEKYSAIIRPLRHYLVTKKMVFKVLAVIWILSTLIGITPLIWKNSQSLPLCFAIYSIVCLVLVFVIPYTFMIYAYIVMFKAITSKRRPSSSHRGVTSRQRRRRTSDRKCILVFALMAAIFAICWLPYFTIMMVLNIKGYLRIRIELTPPIAKAAEVFAFVRYITSVVNPLLYTFFKRDFSRALINILFKSNNRTNLASRQSFLRHRSVTSVQSRLSWTRESSEREKHSVNANLLEEQQVFVSSV

>pdam_00013529-RA protein Name:"Protein of unknown function" AED:0.06 eAED:0.06 QI:0|0|0|0.8|1|1|5|0|669

MILLTIYLEQPQSSRRDTSSGIQYGHATVTSSHMMLICDDVGASWRDLGTQLRLPSAVVRNVENDYTLCRERAWQVLDRWKQRNGTGATLGNLTDALEKIGKRNAAQRLVETEKVEAGLHCARVQSSKLRREVDLLRTGLQNEKQKHDTTVKELREFTKSLQEMLTEKGEDERQHIETRVQGLETTIKRLETYLSTEKTADKEVLMQETTRLGKICDQMEEGLRVVQENIKMISENQKEITGKSLQPENSAWEETSERRSSARQKGGMADRSQKDAGQRYGERQKNEGATIQLLYEGSQACLRCPDLEKELNDIKTKRETIKNKISQLELKHKEDKKKWDEDKKEKDKKIKENELEIGNLKKTNEKQQRQKERLSKKKAEMEKKKTEVMEENKKIMARIEKLLAEKEELEKALLVQTGAKEQERLKAENGMLLTRIEQLSKRVNNSELECKRLHEELXXXXXXXXXXXXXXXXXXXXXXXXXXXXXXXXXXSIIIYTGVFLPTGKEFATNCPGVICNLKRDVTSKLHASRSSDGPGKASDIFDHQKGTTSGTEERKDSWWSIDLGSSHRLVITHYSLRHGKRDGESVLTHWQLEGSNDGMNWKKLETNYKRADPPRFRDPHPYYTGTWSVEGKMGAFRFFRIFQTGRNSSHKYGIYLSGIELFGVLLNI

>pdam_00007837-RA protein Name:"Protein of unknown function" AED:0.25 eAED:0.25 QI:56|0.83|0.85|0.85|0.83|0.85|7|0|885

MASASEVCWSDAALAVCRLVEETKGHKTLKYLGPSCLVHFSDTSTPKILSSTSSGKTSISERASSYFLVASAEVLKKNQIEACENNSGRSVKIVAEFVRVRDGRKLERKTLSPIKVGGELCRALDDIVESDGMIFIALTGLRRSIFNKSNLLSRALEVAEIQGGNSSARMTSNANDLRCVLFTLVTCGKKAFQLQDNPEFGTKVFDLLPFDTVLTCENQTRSSRYFLQNDEKKRFLNEDEFPKNDIPFGAIILKNVMFAGVLNFDSRQPSPVFVESSLQTAIQSGCDEREAERLVGAVGGIYPFLDPMGKERLEANDKTGSYGYPQPTVTPSASDKFVAGFQSTNDNSGNEDFRTIPCTTNQAESDEKAAPIENSCRSLDKSLDSNRVVYVPINLQSADNENQPAGECSAPELSPETGTGGSEEEVQGGPADEQVQGSEEEGGERVTIQKKEEKSVSQGYREPLTAMVPRVTGIQRNPAEIPLAPAVSGGPEYSLPPSPQRCLETQGASTDLNSHGSGFLFQNGRYNELPEGAQGEGWEQSLSGSRHPKKEEHPEVKPSISISTGTKKTYKLADLLKETLLSCLELLEALCRSLDKEYKYGLCKYWKHLAEYFHISEQEYQRFEFQPVFSPTELLFEYLQTADPDVTIGCLKVGLRKIERLDVIDLLVQHEKCDPLALNDETLVSSLFDTDPDIIGELAYLLDIQKSGVKKWSDLAPKLNIPRRIFRMFENCTAGNPTEKVFEIVKVQSPKLTIGELINHLKALKRHDVIKAIKKSTKVTEISVIKELVADVEVMEEVCDLLNQINRTTTVSGLRNLGNRLKIKKEILDDLLPSMEVNQSPTEALIRRLGGSNPSLTLVDFIWALHEISRPDVIVLLDEYLPADT

>pdam_00015331-RA protein Name:"Similar to NPFFR2 Neuropeptide FF receptor 2 (Homo sapiens)" AED:0.18 eAED:0.29 QI:0|0|0|1|1|1|2|0|273

MTFLSENSTQITIHAASSNSDQACEITADSAVEVSAKALAYIFIFMVSFFGNIFLLVVIYKNKQLRRSINYFVFNMAVSDLFNPLTVMTVKIVEIISGSESWKVDRPWLLGNILCKLAYFLPDVSLLVSIGSLLLISIDRLFAVVSPLKAKLISSKVRLISILITWCVAIVFHAPYFYSFKLILYENKTYCTLNWGPALDHLKTHNGYVTASFITFILIPVIILVIVYGIIAWTIRKKNKKTRTIATALSNVFIVTVLVEGYQVCKSTIECER

>pdam_00015337-RA protein Name:"Similar to Npffr2 Neuropeptide FF receptor 2 (Rattus norvegicus)" AED:0.25 eAED:0.29 QI:0|0|0|1|0|0.2|5|0|698

MTVNTTVRYFSKNSTQATSHAASSNSEQACETAADSAVEITAKALAYIFIFLVSFFGNIFLLVVIYKNKQLRRSINYFVFNMAISDLFNPLTVMTVKIVEIISGSSSWKVDRPLLLGNILCKLVYFLPDVSLVISIGSLLLISIDRFIAAVFPLKAKLISSKVRLISILSTWSVAIAVHAPYFYSFKLILDENETYCKLNWGPAFDHMKTHRRFLTATFITFIPIPICVLAGVYSIIAWKIRKKNQKTKEKLSYRQNHRDKQLRKIVRLAMAIMISFVSCMTPLLIYSFLTIFLWNWESPPICAFQTVIPFISVFLLHSWSAVNPCICFIFSENYRNGLRQCFHCNGLGRRKQRSFLNLFHDSRAPLDSIRDKLVTLIYFVIVQCPSDGMVQCELCEERLHMSCEGFKTASEGEWLCIVCRLPDSRRLPNLKEYEVILQRAGLDRLSHEEVKTLPVCARHRHGMGKYWRPSKLCQYSGHKGPPTSVKSRDVINPTMAKEVSQLFDISVPIGSPLDHLKTHKGFVTASFITFTLIPVIILVIVYSIIIWTIRKKNKKSKERLSCRQNHRDQQLRKIVEMTMAIMISFVTCMTPLLTYLFLLIFLWNWKSPPICAFQTIIPFISVFLLHSWSAVNPCICFIFSKNYRNGLRQCFHCDGLSRRLSSLRENYRMRTMTSSYGDSSLEIHSSSVHIISYSRNA

>pdam_00015332-RA protein Name:"Similar to SIFaR Neuropeptide SIFamide receptor (Drosophila melanogaster)" AED:0.21 eAED:0.32 QI:0|0|0|1|1|1|2|0|263

MIANTTVRHFSENSTQTTGHTDSSNFNQACEITADSAVEIAAKALAYISIFLVSFIGNISLLVVIYKNKQLRRSINYFVFNMAISDLFNPLTIMTVKIVEIISGSSSWKVDRPWLLGNILCKLAYFLPDVSLVISIGSLLLISIDRLIAVVFPLKTKLISSKVRLMSILSIWFIAIAVHAPYFYTFKLIRDENETYCKLNWGPAFDHMKTHRRFLTATFITFIPIPICVLAARTTATASGNVFTVMASVTENQVCKTTTKCKR

>pdam_00015336-RA protein Name:"Similar to GHSR Growth hormone secretagogue receptor type 1 (Sus scrofa)" AED:0.43 eAED:0.51 QI:0|0|0|1|1|1|2|0|177

MAVSDLFMPLTIMTVKIVEIISGSESWKVDRPWLLGNILCKLAYFLSDVSLVVSIGSLLLISIERVIAVVFPLKAKLISSKVRLISILSTWFVAIAIHAPYFYSFKLIPYENEMYCIMNWGPAFDHVKTHRGFVTATFITFIPIPICVLAILYARTTAMALSNVFIVTASVEGYQVC

>pdam_00009194-RA protein Name:"Similar to lat-2 Latrophilin-like protein LAT-2 (Caenorhabditis elegans)" AED:0.09 eAED:0.09 QI:0|0.4|0.36|1|0.9|0.90|11|0|493

MSPFNLFVSNADGHCSRPNDTLGPSELNRSVCQQYVRKMPQQWHRMKCGNKTPTSIDKTCCRSWCEQLTSCFSQLPFWKEDCVRMWRCKENKNPRKQISIILKNTTDSSYLGRIKVKQCWARKPNFPTIKTTPSSEFSNKKLVDTILKEVMGSSENDVNQERMERTFLTISDLEEFIGNYAQNHLTQTVPKMYISSENADILVRKIFHDNETGVQFEDDKGENYISLPVTGFDKGSVVLCVIYKDLHKVFLKNQTDSTPLNRFSNILSATIWPRNNTFRKNVTLKFKNLANASNRSCVFWNTSENRWSGQGCLLLSRNDSYTECSCNHLTHFALLMQFDRGTSDNGLTKTDEKALEILTYVGLSFSLVGITLTITSYAVLTDMRGPLSQIRVSLVASLGAGQLFFLTGSGAVENKSACVTVAAFVQYFLMAAFCWMLIEGVYLYLFVVKVYDINDKMKVSHGFSWGLPALVVSISLGIAAGTGPGIKSYVSEK

>pdam_00019552-RA protein Name:"Similar to NPY1R Neuropeptide Y receptor type 1 (Sus scrofa)" AED:0.24 eAED:0.27 QI:0|0|0.25|1|0|0|4|978|573

MDTNASNGNDSFDSSLINFTKAEGFLWFVAFMADLLAKFILNLITIIVFVRQRKLQHRSTYLVIHLVIVDLLVEAVSGPLSLTPPPLTNVSLLYLRSIPSRLFPIASATTLTAISLERLHVTFCPFRHLTVKKRAYVKAIDAIWMISTSLEIIQTFYQCNRSVSSTRVYGHRNREKRLTTSLIIVTFVSLLTLSPVFIYVTIQIFFSVPDLSLSKGSGIRSSILGLKLLNKSINLLHDEFKIPNRPCKDMLSLMAEDHNDSSFNHSFESRNSSVCGSSDCIPWLAVSITECLAIVIFNLLTIIVFVKQRQLQRRSTYLIIHLAVVDLLVGAVSGPVIIAYLFKFYLDSQKKHSVIFPIFSDLFPTTSIANLAVIALERRHATFYPFKYREVKKWIYGIIIVAIWFVPLCREIAQDVLYYREGNSDLSILFSRFSYVLYSSVFLSIICISYIAIFIKIRCSPNPHSHNLGPTKRERRLTTTLLFVTLASLLTWLPSTFFRIAIFWGHRMMSSQSFFYIYVTVAALIGASSLANPIVYAVRMPEFREGLVKLFRKAPNHAYEADSPLRTRQSSLP

>pdam_00023989-RA protein Name:"Similar to trim71 E3 ubiquitin-protein ligase TRIM71 (Xenopus tropicalis)" AED:0.01 eAED:0.01 QI:193|1|1|1|1|1|2|90|812

MASAAPTFLSTKETTNYARLCRLMVDVGTQALRDTFDAIHTPTNLHIVLAGNKATLQTLRTRKVINATQWGKLFPAISTSVSSAQFDITLLMVLLRNLCGLTSPATGWDKLPAVTDLSREADIARVKYYRNTVYGHAERASVDDAAFNAYWDDIRDTLVRLGGVKYKTAIDKLETEVMDPDLEDHYKEVLRQWKKDEDNVKDQLNEVIKKLDDLESSVKGKDSKAEELLHESIKCKEKYHENEVVQYYCQDCNVCICQKCSILIHNRHAMVDIQQTVEEQKMEMKQVFARVKERMAIVNKQIIEQTELMSKSEKEICAAEKKVTEISEEIIRIAREHETAAKTKLEEIKASQKIIYAKKLEEFQVYADQLRNSVECGEDIVQKKVGLKILQAGNTVVARCEELLTSKDIEIFNPQSVIYRVNAESLNTVRHLVPGQVIASYTDPSKSAAEGNGLQEAEIGAEIGFTVTTRDSEGKLFYDEEDRVTVKIRSPKGEDGEIKPKHYQNGHYTVRYEPKSIGLHEIVVEVNGKPLTGSPWRVQVTAHQYKTLRSFGSHGKGPAEFVRPGSIAVSERTGNIAIADSVNKRVQLFDSKWKYLRAIGDKGPVAERIAYPNSVAFTASGDVVVIHNETGQPGEMLLFTEHGQFIKHTGQYLIDPKSVSIRSDGHMIVCDSGDKSVKVLTPDGSGLKQSFNALDCDTLPIHAVYHEDKFFVSYVMANCVKVFNKRGELQYDIGREGSGDVQLNCPVGLTIDRCNNLIVCDTGKSRVRVFSLDGTLVNSFSEGMDFPIFVAVTKDSELLISDFFKNVIRVFQ

>pdam_00014397-RA protein Name:"Similar to ADGRD1 Adhesion G-protein coupled receptor D1 (Bos taurus)" AED:0.16 eAED:0.16 QI:315|1|1|1|0.61|0.72|22|328|889

MFDPQNRHFVHSWEMDCPVKKVYRELWRNVPGSSVADLKGSNFYPCAASKTEFLDTFCLYKSYQDNSYGQRLRSFFRAPETGHFTFQTSCDNSCQLWMSSSESPAGKRLIIDQRVSSQAHSWDGSVNQLSGKIHLEKGKLYYMEVLHKEDRLADFICVGAKFPTLKKEQPISSRHLVMNRTELQLMGCKGNIQQHNVECQEDTSCESPHQKLNKFKQSMDSSMRSLRSISADGSPSSLKQIKQVTKESTEIINRILEDSKKEEWRVSNMTSAMEIAEITETFGKELAHKWSNDTSGMVSLDANIALQASVLPSNSYKFSVAEFDVNWRGIKDILALKITKGRPDNKTKVFSVIYKNLHELLPSNPEKIIEKQAFEEKYLELNSRIISSLIYPPADSQTLEVRVTFQHLKHKSNASLTPVCVWWDFAAAGVNGSGFWSTKGCQVDEATSNETHSTCKCNHLTNFAVLMQVQSDSDLEVNKSHKIALQIITYVGCGLSLIGVTLTVFIIAAFSGLRSERNLIHLNLSLSIGTFQIIFLAGIETTSYEIACTVVAVLLHYFLLVSFSWMLVEGVYLYLMVITVFENNKEQLRIFGACSYGLPGIIVLISAIVAHDGYGTDLSCWLSTAKGVIYAFVGPALAIILVNAVILIMVIREIIKVQTNGVSHSTKFDLIKSGFKSTVVLFPLLGVTWLFGILALDRNTIAFQYLFALCNSLQGFFIFVFHCLLNSEVRKVIQRKKEIWSSRRTTLFSPSTTTPSSAVAASTSHGTTLSDVNMISVSDDASNRKLATGSGNSKSKLLPKENGNDASQNANDRVPVSTDGANKGLSMENSSPAWLSKTEHDPGPKRIKLPPVTVPENAKRKQKRITNKNLKQGSKGKLNTAIEPLQEET

>pdam_00020431-RA protein Name:"Similar to ADGRD1 Adhesion G-protein coupled receptor D1 (Bos taurus)" AED:0.21 eAED:0.22 QI:0|0.56|0.47|1|0.68|0.76|17|0|699

MSLIYVQVSNADGHCSYPNDTLAPSELNYSDCQRHLDKMRQFWHRMECGNETPTSIDKTCCRSWCRQLASLFSQTSSWRDRCFKMWGCRENEKARMPFSIILTNTTDSSYLGRIKVEQCLTLNFPAIKLTPSSEFSNKKLVDTILKEVTNSSGNKVTQEEMERTFLTVTDLEEFIGNYAQNHLNQTVPKIDIRGEHADILVRKIFHENETRVELEDDKGENYVSVLVTGLDNGSVVLCVIYKDLHEVFLTGQTDSAPVTKISWSGQGCLLTSKNECYTECSCNHLTHFAVLMQSDWGTSINFISKTDETALEILTYVGLSFSLVGITLTIISYAVLTMRRREAACFGILQKTGQGCLLTSRNQSHTECSCNHLTHFAVLMQFDRGTSGIVLTKTDEKALEILTYVGLSFSLVGISLTIISYAVLTDMSGPLSQIRVSLVASLGAGQIIFLTGSGAVENKSACVTVAAFVQYFLMAAFCWMLIEGVYLYLFVVKVYNIDDKMKVSHGFSWGLPAVVVSISLGIAAGTGPGIKSYISANFCWMSSSNGMIWIFVVFVVLIELLNTLILVRVIKEMKNMQHAKDKISEQMRLGVRACVLMIPLLGITWLFGLLSPMHKAFAYIFTIFNSTQGFLIFLLHCVKNSKIRSRFKRRITGATLTAAEIRGIKRAAQVNEVINVILPRKINVQPRNNRESGLEISEI

>pdam_00020432-RA protein Name:"Similar to ADGRD1 Adhesion G-protein coupled receptor D1 (Bos taurus)" AED:0.31 eAED:0.32 QI:0|0.55|0.47|1|0.66|0.73|19|0|648

GHCFRPNDTLGPLELNPPGCQQHVQETLQYWHRMKCGNETPTSIDKICCSSWCEQLTSCFSQERSWKEDCIRSRFKRRINAVNPTADEVTGVKRASQVNETISVILPRKLNVQPPNNHENSSYLGRIKVKQCWARKPKFPAIKLTPSSEFSNKKLVDTILKEVIGSSDNNINQEKMERTFLTVTDLEEFIGNYAQSHLNQTVPKIDIRSEHADILVRKIFHENETEVQLEDDKGENYVSLPVTGFDKGSVVLCVIYKDLHKVFLKNQTDSAPVNNFSNILSATIWPRNNIFWKNVTLRFRNLADAPNRSCVFWNTSKNSWSGKGCLLTSTNESYTECSCNHLTHFAVLMQFVKGTSSNVLTKTDEKALEILTNVGLSFSLVGITLTIISYAVLTDMRGPLSQTRVSLVASLGAGQIIFLAGIGAVENKSTCVIVAALVQYFLMAAFCWMLIEGVYLYLFAVKVYNINDKMKVSHGFSWGLPAVVVSISLGIAAGTGPGIKSYVSETFCWMSSSNGMIWIFVVFVVLIELLNTLILVRVIKEMTNMQHAKDKIIEQIRLGVRACVLMIPLLGITWLFGLLSPLHKGFAYIFTIFNSTQGFLIFLLHCVKNSEIRSRFKGRINGVTPTTDEVIGIKRAAQICVYLKLRKF

>pdam_00023069-RA protein Name:"Similar to roco5 Probable serine/threonine-protein kinase roco5 (Dictyostelium discoideum)" AED:0.05 eAED:0.05 QI:134|0.83|0.76|0.92|0.91|0.76|13|0|1356

MDFSETQEQEENHFVQDEDEIDIKCLLKEGKPTEGDIDWIVREINSNPDEKKKLFELLRVNDDDPGYNSGDSIDNAKDFTDGMTIPRHLLKNQTYEELAALLGESPLNRRDLAVKTCCIQKDLLPTYILARGNQAVTAYRGALTSGETLDNRVKVILIGEGQVGKTSVAKALRGEKFEENERSVNGILMSEVIKNATHDKPWGNSATRENARPVSSQTNGINAIEGISLEDEVDGARSNSPEIELVVWELSGQGAYRAIHPMFMTPDAVYVLVFDLSKGLFDQATAKDNENGSATNSENEDSNLDCIMRWMDILNSMRYSAADEILPPVLLVGTHADCVNGNPRSIMDALLDRFRKIELGDQIKGNFVLDNTLNVPGQEDPQVAKLRRRILDEANKLPHAMKKIPLKWFDIERKIQEEAKKGIKYYTTKEVLTKKVCQTQDLNEIDQILLFLCDRRSIIYHENASKQDGLVVLDPQWLVGRLCKLLSLPPEEEDKIEFRNLRKELRETGILHQKLLHRTCTEWEVKDIEEALVSLMKHYNLLFHCPRKEKNPIYLVPCMIKRATGENEVIPSKNGSSTSPPIYLIFGTKFVPIGLFSRLAMLIGAWAAKKSSFEQLQINADTACFILDGVNFLELKCFKSVIQVQVWSEDSTRQYSDPGLYSEIYCCLERSLKAVCATCHWLHSVSWDLSVQCRLCAGMVNLKTRKCIWHDKRNCHHEDCAHYIPLKRHIALFCQQAMKRLPEEKFERWVQASGKNNSQEETGKENIASTNEITVLLLAEEWGSGKGGLSTINRQLAIELARYSNLRIYFYVIKSNEEDKQMAKSSNINIIKAKKRPGYNNGFERLSFPPEELPLIDVVVGHGVKLGKQAQLIKVKHPRCVWVQFEHTAPEELGTYKNYEGKIARGESKHRDEVDLAKRADVVAAIGPKLTEYYKTSLRPDKGGPEKVFEMIPDPSMFHEFRPCKQVKEDRENFNVLIFGRGDSEDFKLKGFDIAARAVAELNDEKYHLYAVGAPHGQQEDVEKKLLQCDISRRQLTVRGFIECRDEVAQLLCEVDLAIMPSRTEGFGLTALEALAAGLPILVGKNSGFAKALQKINESACIVNSEDSKEWANAIRFIKEKPRSERLQEAKRLKERCESEFAWSRRCQALTEKILLLVEKKSHNKDMKGEQRCEVAAVPEGQEEDETQNQSQTTRRPDAVEEQEPTGGDEGYSSSRDRTNSTEFSSGHEETSNSPNAVLNVRRNFHADTEDTRLNEQQNGSQLERDENQETGTSQKKYVFNSRTTAKEKSSESKGLRRRDDERDDVFKYQGPAVLENKELLDTSYISNVNSNSIMLSKVEFYEGLLECASAKRFYS

>pdam_00026002-RA protein Name:"Protein of unknown function" AED:0.08 eAED:0.13 QI:65|1|0|1|1|1|2|0|224

MKLGRRLGVKNPKLQDIKQRYEELSERGYHMLMHWKQENGCKATYQILNSALRHKLVQRKDLAEQICYNHDIPDVPRNHFDVEVCRRELAEHYKRTAKVPTSVWSKICAVNIHEIYTRLSWVKAEQTPAGSSRAVLNHYTDVFAENKNGLLSNRILVQGETGIGKSTFVKKLAIDWAELDENRLTDEQRAILKKFELAVIIDLKKVSKYQNLRDIISASHIFAD

>pdam_00013768-RA protein Name:"Similar to mshA D-inositol 3-phosphate glycosyltransferase (Corynebacterium aurimucosum (strain ATCC 700975 / DSM 44827 / CN-1))" AED:0.24 eAED:0.25 QI:0|0.5|0|1|0.5|0.6|5|0|1161

MEKCRLNITLATGAVGWNTLVNNQLLIELAKNSKVKVTGLVPRSTQEQKEQAKKLNVELVDGQELDGFSSMELLSFPPDDLEIDVLFMHSYGLHEGKHAQNIRTAKKCKWFHIIHTIGEELAKYMDNKCEQESEHQLQLKLSRKADCVIAIGPKVADTFRSELCHCGKSNDVIDLTPGIMKELVGVRAFQQCGEIFRILISATWFAKYYKVKGLDIAAKAMTFLKEVSCHLIYIVNKDEDTEDLKNQLLQEGIELNQLTVRRLQSSNLENWKSQLCQVNLIIMPARTEGFGTTNLRAISADLPVLVSQNCGFGMALKKLPSGEKHVIKSNKPEVWAERIKEVLEKRPEDLMSEAKKLRKEYEEKYNWEKQCEDLVDRMLTMFPSKQEDFKQCVEHGEESDQARGGDPEVQQCETEVMVYEERMHENGNLGSAELRLAGKKKINQSERKANKEMVDVLQSRTQELSLVTRESESKSIDYKEKVEIVQERTQEVGHAAAKEVKKKGSGFDQTDGAAATLDGVNDSESNRDQTGQPVKAQDIPYSILSKICLKLNAKDDLSFRDFRLLGEKMGFDKDLTKVLEQKSNPTYELFQLWHPNPKSTVENLLMILKDDEMGRWDVTGWKKKGLRIISKLNVTLASDVDGWNKTVNGQLVIELGRNDRLCVTGFVPKNTQQQREHARSLNIQLVESKDHDGFSSAELLCFPPDNLNIDVLFIHSHGTNLGRQAQAIKDLKKCKWVQVVHSFHEECHTARYENENELQRKMCEKADAVIAIGSKTAESCRRALRYSLKQENVINLTPGVCEDLIGVRRVYEDLATFHVLISGSLSHFSIKGCDVAVKAIKLLNTPSYHLTVVVKSHDETAGITKALLNEGINSQQLDVCVAGSREEWRKLMCKVDLAIKPSRNEGFGMSGLLAISADLPVLISEHCGLGMVLKTVLFGNSHVVDSVKPEVWADRIQQVRDKDPELRREEAKQLRDGYTQKFNWKNQCEKVVETLCKLGQKIEGPTEKQDQEEAVMKLTKTVEKTNLSDESSTREEKDTKMKQDVVDAGRSLANERLSMSVRNLPLPVYSRVCLSLNVLRDVRWDDFRMLGEKVGLSRDEIDFIQQQRNPTDEILKTWSSNPHEATVAKLIELLKHKDFRRMDVAQILEEWVSEMNEKYNN

>pdam_00013766-RA protein Name:"Similar to mshA D-inositol 3-phosphate glycosyltransferase (Mycobacterium gilvum (strain PYR-GCK))" AED:0.28 eAED:0.28 QI:0|1|0.5|1|1|1|2|1206|572

MTEPCVIYSLAFSIAFPGSLVQDPNKLNVTLATDAEGWDKSVNEQFLLELAQNPQVRVTGFVPKHTPKQKDHARDLNIELVDAKEITGFPAVELLAYPPDDLDIDILIIHSYGRDLGRQAQIIKDKKKCQWVNVVHTVSEDLEKFSVKKSADSAANDQLTEHDLQTRLCEKSDLIIAIGPKVTEAYRAALKYSGKDQSVICLTPGIIEEFLGVRGEIKGSGEIFRVLLSASSKYFLVKGCDIATKAINLLKDSSYHLIFVRQPKDNEDQLKKAFLDLGINQHQLTFRSSGSKDFWPRLLCEVDLVIKPSRTEGFGISGLRAISADVPVLVSGNCGLGMVLKSLPSGAQHVIDSEDPQDWADAIKKVRAKAVQVRKAESEQLRNDYMQQFNWKMQCNEIVKKMFSMNPHKSEYRKKAVEWQFDVADTTRDLQSLQVNRDDVDGGPYYQYETEKMSIQETSQFPRTYKRGDISRRNMGVSNLPLKEYRKVCIMLNTKRDLKFDDFRMLAEKVGFNRDEIRMIEQGENPTDKVLQTWSSKREATVGNLIDLLTGEDLERMDVAKVLEDWVNDTCH
